# Functional Attributes of Myco-Synthesized Silver Nanoparticles from Endophytic Fungi: A New Implication in Biomedical Applications

**DOI:** 10.3390/biology10060473

**Published:** 2021-05-27

**Authors:** Prabu Kumar Seetharaman, Rajkuberan Chandrasekaran, Rajiv Periakaruppan, Sathishkumar Gnanasekar, Sivaramakrishnan Sivaperumal, Kamel A. Abd-Elsalam, Martin Valis, Kamil Kuca

**Affiliations:** 1 Department of Biotechnology, Bharathidasan University, Tiruchirappalli 620024, India; drspk1989@gmail.com; 2 Department of Biotechnology, Karpagam Academy of Higher Education, Karpagam University, Coimbatore 641021, India; kuberan87@gmail.com (R.C.); rajivsmart15@gmail.com (R.P.); 3 School of Materials and Energy, Southwest University, Chongqing 621010, China; sathegene@gmail.com; 4 Plant Pathology Research Institute, Agricultural Research Center (ARC), Giza 12619, Egypt; kamel.abdelsalam@arc.sci.eg; 5 Department of Neurology of the Medical, Faculty of Charles University and University Hospital in Hradec Kralove, Sokolska 581, 50005 Hradec Kralove, Czech Republic; martin.valis@fnhk.cz; 6 Department of Chemistry, Faculty of Science, University of Hradec Kralove, 50003 Hradec Kralove, Czech Republic; 7 Biomedical Research Center, University Hospital in Hradec Kralove, Sokolska 581, 50005 Hradec Kralove, Czech Republic

**Keywords:** endophytic fungi, silver nanoparticles, antibacterial activity

## Abstract

**Simple Summary:**

Greener fabrication of silver nanoparticles has received extensive attention owing to their low cytotoxicity, tunable surface features, and stability. Fabrication of AgNPs using natural entities has received substantial consideration as an alternative to toxic chemicals. In this study, we have screened the potential of an endophytic fungi *Penicillium oxalicum* strain LA-1 for the synthesis of AgNPs and analyzed their potential multipurpose values in biology and medicine.

**Abstract:**

To develop a benign nanomaterial from biogenic sources, we have attempted to formulate and fabricate silver nanoparticles synthesized from the culture filtrate of an endophytic fungus *Penicillium oxalicum* strain LA-1 (PoAgNPs). The synthesized PoAgNPs were exclusively characterized through UV–vis absorption spectroscopy, Fourier Transform Infra-Red spectroscopy (FT-IR), X-ray powder diffraction (XRD), and Transmission Electron Microscopy (TEM) with energy dispersive X-ray spectroscopy (EDX). The synthesized nanoparticles showed strong absorbance around 430 nm with surface plasmon resonance (SPR) and exhibited a face-centered cubic crystalline nature in XRD analysis. Proteins presented in the culture filtrate acted as reducing, capping, and stabilization agents to form PoAgNPs. TEM analysis revealed the generation of polydispersed spherical PoAgNPs with an average size of 52.26 nm. The PoAgNPs showed excellent antibacterial activity against bacterial pathogens. The PoAgNPs induced a dose-dependent cytotoxic activity against human adenocarcinoma breast cancer cell lines (MDA-MB-231), and apoptotic morphological changes were observed by dual staining. Additionally, PoAgNPs demonstrated better larvicidal activity against the larvae of *Culex quinquefasciatus*. Moreover, the hemolytic test indicated that the as-synthesized PoAgNPs are a safe and biocompatible nanomaterial with versatile bio-applications.

## 1. Introduction

Innovative materials with superior optical, physical, and electronic properties will always find a large spectrum of applications in physics, chemistry, and the biological sciences [1]. In this context, nano-sized particles (<100 nm) are receiving a wide range of attention and possess diverse applications in solar cells, batteries, electrochemical industries, pharmacy, and medical fields [2]. In recent decades, metallic nanoparticles (silver, gold, copper, iron oxide, and platinum) have gained significant interest because of their surface-to-volume ratio, tunable size and shape, and ease of synthesis [3]. Among metallic nanoparticles, silver nanoparticles (AgNPs) are preferred over other metallic nanoparticles, as AgNPs exhibit superior optical and conductive properties, which find an extensive array of applications in developing antibacterial agents, optical sensors in industrial and household objects, healthcare-related products, cosmetics, medical device coatings diagnostics, orthopedics, drug delivery, and in the food industry [4]. 

Cancer is one of the most lethal illnesses in today’s world. Breast cancer has been a leading cause of death in women. According to the WHO report, 16.3 million deaths from such diseases are predicted by 2040 [5]. The most common cancer treatment and control methods are surgery, chemotherapy, targeted therapy, immunotherapy, radiation therapy, and hormone therapy. However, these traditional therapies have drawbacks such as drug resistance, recurrence, metastasis, and toxicity [6,7,8,9]. Nanotechnology advancements in medicine have given rise to new hope in the biomedical and medicinal sectors.

Similarly, the “war” among humankind and infectious pathogens has been going on for a long time, and pathogenic bacteria have caused many deaths in humans. Besides discovering the first antibiotic, penicillin, a phased breakthrough in combating diseases was accomplished. However, bacteria have evolved and developed resistance genes due to widespread antibiotic misuse, resulting in resistance to available antibiotics as well. As a result, the study of alternative antibiotics is inevitable [10,11]. To accomplish this task, researchers are concentrating on Ag+, which has a high antibacterial capacity due to its increased interaction with pathogenic bacteria.

Various physical and chemical methods have been developed to fabricate AgNPs, however, the concern remains such that the fabricated materials are expensive, hazardous, and toxic to the human beings [12]. Despite the pros and cons of AgNPs synthesis methods, biogenic nanoparticle synthesis will substitute conventional nanoparticle synthesis [13]. The plants and microbes have inherent ability to accumulate and transform metal ions into nanoparticles by their active metabolites. This unique property has made plant crude extracts potential nano-factories to synthesize various metallic nanoparticles [14]. Similarly, the microbes are also well known as possible systems to synthesize metallic nanoparticles. Microbes like actinomycetes, fungi, bacteria, viruses, and yeast are rich resources for nanoparticle synthesis [15]. 

Specifically, fungi are useful secretors of various intra/extracellular enzymes because of their toleration and metal bioaccumulation capability. Their facileness in scale-up, economic livability, and facility of employing biomass are some of the merits of the exploration of fungi to synthesize metallic nanoparticles [16]. Numerous studies have reported the utilization of fungi in synthesizing metal nanoparticles via reducing enzymes intracellularly or extracellularly. The reported fungal species inferred that the use of fungal biomass or cell-free extract yielded nanoparticles with significant size and shape [17]. 

Endophytic fungi inhabit leaves, stems, flowers, seeds, and fruit without destroying the plants [18]. Moreover, endophytic fungi produce an array of secondary metabolites with necessary usefulness in medicine, pharmacy, agriculture, and environment [19]. Our previous report proved that the endophytic fungi *P. oxalicum* LA-1 strain can produce hamisonine, a limonoid compound exhibiting excellent inhibition activity against III instar and IV instar larvae of *C. quinquefasciatus* [20]. Furthermore, to explore the efficacy of endophytic fungi *P. oxalicum* LA-1 strain, we have synthesized AgNPs using the culture filtrate and assessed its efficacy against human pathogens and mosquito vectors.

## 2. Materials and Methods

### 2.1. Chemicals and Reagents

All the chemicals and reagents used in this study were of analytical grade, purchased from Sigma-Aldrich (St. Louis, MO, USA) and Himedia (Mumbai, India). 

### 2.2. Isolation and Characterization of Endophytic Fungi

Our previous report provided a detailed methodology for the isolation and characterization of the endophytic fungi *P. oxalicum* LA-1 strain from the medicinal plant *Limonia acidissima*. The isolated strain was sequenced using the internal transcribed spacer (ITS) region and submitted in GenBank with the accession no: KX622790 [20].

### 2.3. Preparation and Extraction of Culture Filtrate from P. oxalicum LA-1 Strain

The fungal biomass was prepared by growing fungi aerobically in potato dextrose broth (PDB) and was incubated at 24 ± 1 °C for 1–7 days. After incubation, the fungal mat was washed twice with double-distilled water, transferred to a flask containing 100 mL of sterile distilled water, and kept under an orbital shaker at 140 rpm for 48 h at 24 ± 1 °C. After 48 h, the culture filtrate was filtered through Whatman filter paper no. 1, and the extract was stored for further use.

### 2.4. Natural Products Analysis by GC-MS

GC-MS detected the active constituents present in the LA-1 strain culture extract. For the analysis, an Agilent GCMS apparatus, GC: 7890A, MSD5975C-HP-5 capillary column (30 m–0.25 mM, ID, a film thickness of 0.25 mM) coupled directly with single quadrupole-MS was used. Furthermore, the mass spectrum interpretation was conducted using the National Institute and Standard Technology (NIST) library. The compound name and molecular weight of the obtained spectrum were identified. 

### 2.5. Fabrication of Silver Nanoparticles Using Culture Filtrate of P. oxalicum LA-1 Strain (PoAgNPs)

For the fabrication of PoAgNPs, aqueous filtrate of *P. oxalicum* LA-1 strain was challenged with aqueous silver nitrate solution. Briefly, for the synthesis of PoAgNPs 10 mL of the filtrate was mixed with 90 mL of 1 mM AgNO_3_ and the mixture was incubated at room temperature for 1 to 3 days. 

### 2.6. Characterization of PoAgNPs

After the incubation, a change in the reaction’s color formation implied the preliminary confirmation of PoAgNPs. To characterize the PoAgNPs, sample preparation and processing were carried out according to our previously established methodology [21]. UV–vis spectrophotometry was used to measure the surface plasmon resonance (SPR) of PoAgNPs, which was operated at wavelengths of 300–700 nm using JASCO V-650 UV–visible spectrophotometers. The plausible functional groups present in the filtrate may act to reduce, cap, and stabilize PoAgNPs. This was ascertained by employing Fourier transform infrared spectroscopy analysis using a Jasco Fourier transform infrared spectrometer performed in the range from 500–3500 cm^−1^_._ X-ray diffraction analysis (XRD) analysis was carried out to probe the crystallographic structure and orientation of PoAgNPs using the Ultima IV X-ray powder diffractometer with CuKα radiation (λ = 0.1546 nm) (Rigaku Ltd., Tokyo, Japan). Transmission electron microscopy (TEM) exclusively characterized the morphology features (size and shape) of PoAgNPs using a JEOL 3010 transmission electron microscope. Zeta potential measurements evaluated the surface charge of the synthesized PoAgNPs by using a Zetasizer Nano (ZS 90) instrument. For DLS analysis, synthesized PoAgNPs were sonicated for 10–20 min to disperse the nanoparticles and were analyzed using the Malvern Zetasizer Nano-ZS90 analyzer.

### 2.7. Hemolytic Activity of PoAgNPs

In a sterile lithium heparin container, blood was collected from healthy volunteers. Red blood cells (RBCs) were then separated using Ficoll density gradient centrifugation (1500× *g* rpm for 10 min at 4 °C). Next, the RBCs were diluted in 20 mM HEPES buffer saline (pH 7.4) to produce a 5% *v/v* solution. One mL of diluted PBS was transferred to Eppendorf tubes containing 1% Triton X-100 (positive control), RBC saline (negative control), and different concentrations of PoAgNPs (25, 50, 75, and 100 µg/mL), which were then added to the tubes; then, the samples were incubated. The samples were centrifuged at 12,000× *g* RPM at 4 °C, and the supernatant was transferred to 96 well plates. The absorbance was measured at 570 nm [22]. 

The percent of hemolysis was calculated as follows:

$$Hemolysis\;\%=\frac{\left(OD\;value\;of\;sample-OD\;value\;of\;negative\;control\right)}{\left(OD\;value\;of\;positive\;control-OD\;value\;of\;negative\;control\right)}\times 100$$

### 2.8. Antibacterial Activity of PoAgNPs

The antibacterial activity of PoAgNPs was evaluated against human bacterial pathogens. The Gram-negative stains *Klebsiella pneumonia* (MTCC-530), *Vibrio cholerae* (MTCC 3906) and *Escherichia coli* (MTCC-1687), and the Gram-positive strains were *Micrococcus luteus* (MTCC 1809), *Mycobacterium smegmatis* (MTCC-994) and *Bacillus subtilis* (MTCC-2387) were used. For the study, bacterial cultures were inoculated and grown overnight. A 6-mm diameter well was made in Mueller–Hinton agar (MHA) media plates using a borer cork. After the incubation hour, the bacterial cultures were spread uniformly throughout the media plate by using a cotton swab. Different concentrations of PoAgNPs (10 µg/mL, 20 µg/mL, 30 µg/mL, and 40 µg/mL) were added to the MHA plates and allowed to stand for one hour for the perfusion of PoAgNPs into the medium. Finally, the petri plates were incubated at 37 °C for 24 h. The zone of inhibition (ZOI) was measured around the wells in diameter (mm) using a meter ruler. The assay was carried out in triplicates with antibiotic streptomycin as a control [23]. 

#### 2.8.1. Determination of Minimum Inhibitory Concentration (MIC) and Minimum Bactericidal Concentration (MBC) of PoAgNPs by the Microdilution Plate Method

The minimum inhibitory concentration (MIC) was performed in 96 well-rounded bottom microtiter plates using the microdilution method [24]. The bacterial strains were cultured in Mueller–Hinton agar and the turbidity was adjusted to a 0.5 McFarland Standard for analyze the MIC. Briefly, One hundred µL of sterile Mueller–Hinton broth was added to the 96-well plate. In the plate, the first two rows were served as a growth control. For analysis, PoAgNPs were serially diluted in dimethyl sulfoxide (DMSO) to create a concentration sequence from 100 to 3.12 µg/mL and added to the wells having previously inoculated bacterial cultures. Finally, 10 µL of Resazurin dye was added to these wells and incubated for 24 h at 37 °C.

The MBC values were defined as the least concentration of antimicrobial agents that prevent the growth of the organisms. The MBC test was performed by plating the suspension from each well of the 96-well plates in sterilized MHA plates followed by incubation for 24 h. 

#### 2.8.2. Fluorescence-Based Study of the Bactericidal Activity of PoAgNPs

A log phase culture of bacterial pathogens *K. pneumonia*, *V. cholerae*, *E. coli*, *M. smegmatis*, *M. luteus,* and *B. subtilis* was centrifuged at 4 °C, 10,000× *g* RPM for 5 min and suspended in phosphate buffer saline (PBS). The supernatant was discarded, and the remaining bacterial cells were resuspended in 5 mL of PBS. Next, 100 µL of 100 µg mL^−1^ PoAgNPs were added to the bacterial suspensions, incubated for 1 h, and stained using 100 µL of fluorescent dyes (AO/EB) for 15 min. After rinsing the stained sample with PBS, the images were observed using an OLYMPUS DP27 fluorescence microscope. The control assay was performed without any PoAgNPs treatment.

### 2.9. In Vitro Anticancer Activity

#### 2.9.1. Cell Lines 

The human adenocarcinoma breast cancer cell line (MDA-MB-231) was obtained from the National Center for Cell Science (NCCS), Pune, India. The cells were maintained in a DMEM medium supplemented with 10% FBS (Sigma-Aldrich, St. Louis, MO, USA) and with 100 μg/mL streptomycin as an antibiotic (Himedia) at 37 °C and 5% CO_2_ atmosphere in a CO_2_ incubator (EC 160, Nüve, Ankara, Turkey). 

#### 2.9.2. MTT Assay 

The viability of PoAgNPs-treated MDA-MB-231 cells was measured using the MTT assay [25]. For this, the human adenocarcinoma breast cancer cells (MDA-MB-231) at 1.5 × 10^6^ cells/well were seeded in 96-well plates to treat with different concentrations of PoAgNPs (10–100 μg/mL) and incubated in a CO_2_ incubator for 24 h. After incubation, 20 μL of MTT solution (5 mg/mL in phosphate-buffered saline) was added. After 4 h, the purple formazan product was dissolved by adding 100 μL of 100% DMSO to each well. The absorbance was monitored at 570 nm (measurement) and 630 nm using a 96-well plate reader (Bio-Rad, Hercules, CA, USA). All the assays were done in triplicate, and the results were given in mean ± SD

$$Percentage\;of\;inhibition=\frac{(OD\;value\;of\;control-OD\;value\;of\;Sample}{OD\;value\;of\;control}\times 100\;$$

#### 2.9.3. Dual Staining Assay (AO/EB Staining)

The cell suspension of the control and the PoAgNPs-treated cells containing 5 × 10^5^ cells were added with 20 μL of Acridine Orange (AO) and Ethidium Bromide (EB) solution (3.8 μM of AO and 2.5 μM of EB in PBS) and examined with a fluorescence microscope (Olympus, Japan) using a UV filter (450–490 nm). The staining declared the cells, the nuclear morphology, and the membrane integrity; morphological changes were also observed and photographed [25]. 

### 2.10. Larvicidal Activity of PoAgNPs

The methodology was adopted from our previous report [26]. Briefly, different concentrations of PoAgNPs (0.31, 0.62, 1.25, 2.5, 5, and 10 ppm) were prepared. Twenty-five numbers of II and IV larval stages of *C. quinquefasciatus* were introduced into the beaker containing 249 mL of water and 1 mL of above mentioned concentrations of PoAgNPs and kept in an environmental chamber at 25 °C with a 16:8 light/dark cycle. Mortality was noticed after 24 h post-exposure. During the experiment, no food was provided to the larvae. We performed three replications to validate the results. The control (water) was maintained to assess the natural mortality of the mosquito larvae within the test period.

The percentage of mortality was calculated by using the formula:

Percentage mortality (%) = ((X − Y)/X × 100)

X—the number of live larvae introduced; Y—the number of live larvae treated.

### 2.11. Statistical Analysis

Experiments were carried out using a completely randomized method, and the results were shown as mean ± SD (standard deviation). The SPSS software package 16.0 edition was used for statistical analysis (SPSS Inc., Chicago, IL, USA). One-way ANOVA (post-hoc (Tukey’s test)) was performed to know the statistical difference in antibacterial studies. On the other hand, the larval mortality data was subjected to Probit analysis to calculate LC_50_, LC_90_, the 95% confidence interval (upper confidence limit) and the lower confidence limit, the Chi-squares intercept, the Chi-square test, the linear regression, and the F- and R-values. 

## 3. Results

### 3.1. Biosynthesis of Silver Nanoparticles from Endophytic Fungi P. oxalicum Strain LA-1 (PoAgNPs) 

**Figure 1:**
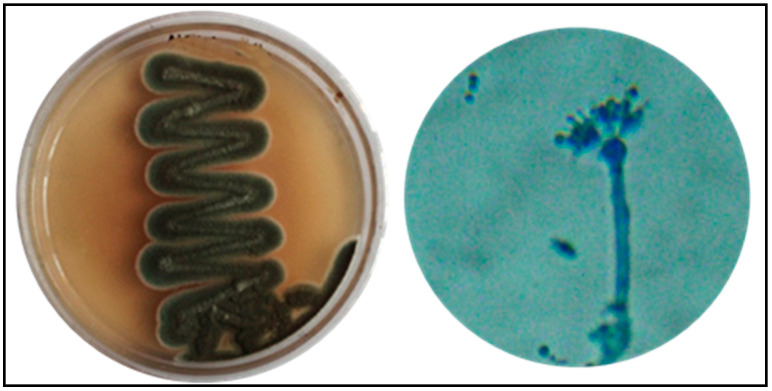
Morphology of *P. oxalicum* LA-1.

In this study, the biosynthesis and the optimization of silver nanoparticles from extracts of the endophytic fungi *P. oxalicum* strain LA-1 (Figure 1) isolated from the medicinal plant *L. acidissima* were carried out. 

### 3.2. GC-MS Analysis of Bioactive Compounds from Endophytic Fungi

**Figure 2:**
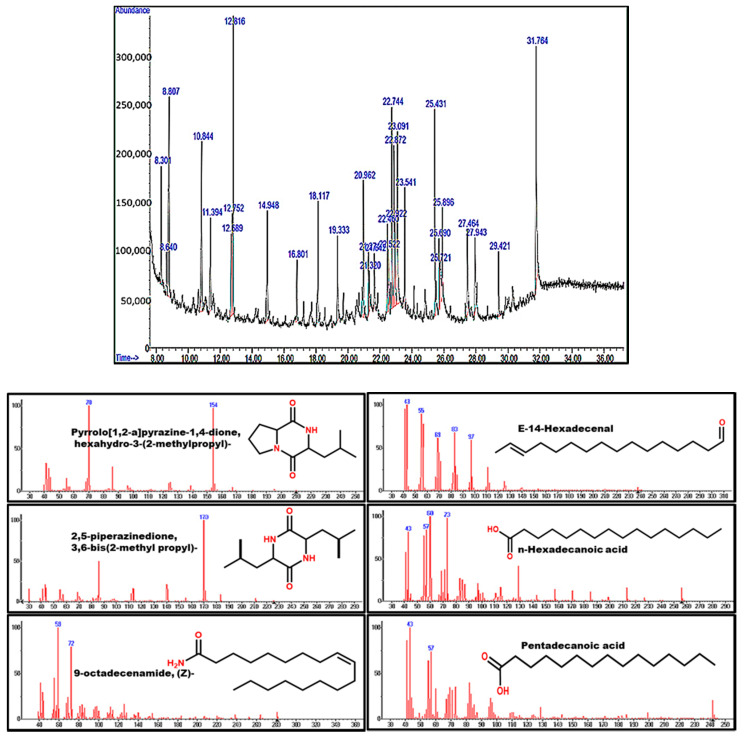
GC-MS chromatogram for ethyl acetate extract of *P. oxalicum* LA-1.

The bioactive compounds present in the LA-1 culture filtrate were examined by GC-MS analysis. Based on the elution order, in the HP-5MS column, the compounds were identified and characterized. In Figure 2, the spectrum for LA-1 extract is provided. We annotated the spectrum with the NIST database and identified 28 compounds present in the extract, as represented in Table 1. Among the identified compounds, four compounds possessed the potential pharmacological activities (pyrrolo[1,2-a]pyrazine-1,4-dione, hexahydro-3-(2-methyl propyl); E-14-hexadecenal; 2,5-piperazinedione, 3,6-bis(2-methyl propyl)- and n-hexadecanoic acid) (Figure 2).

### 3.3. Optimization and Characterization of PoAgNPs from Endophytic Fungi

#### 3.3.1. UV Vis Spectroscopy Analysis

**Figure 3:**
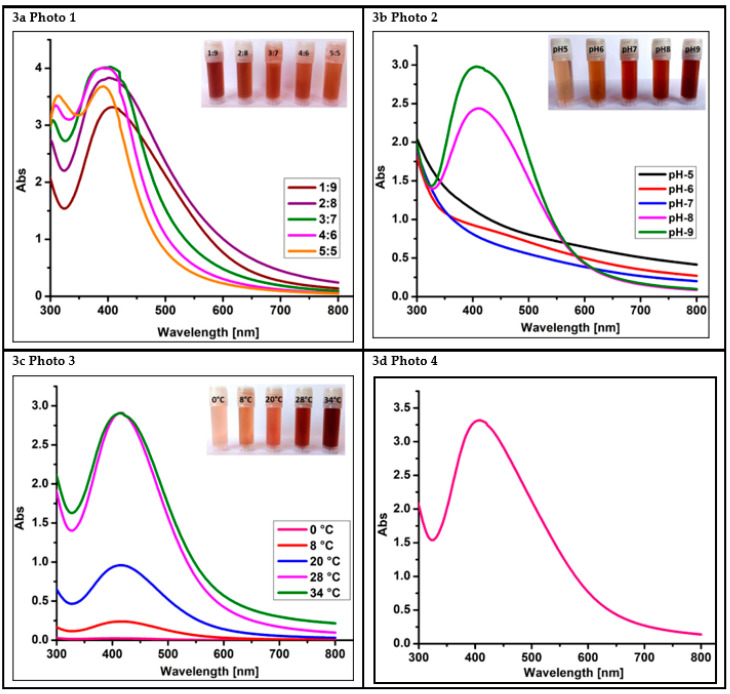
Color change formation and optimization of PoAgNPs. (**a**) UV absorption spectrum of PoAgNPs (fungal extract vs. silver nitrate). (**b**) Effect of pH on the synthesis of PoAgNPs. (**c**) Effect of temperature on the synthesis of PoAgNPs. (**d**) UV–visible spectroscopy produced intense SPR spectra at 410 nm in optimized conditions.

When aqueous culture filtrate is mixed with silver nitrate solution, the prompt reaction is initiated. The reaction gradually proceeds with a color change from white to a dark brown color within 24 h at 37 °C. The optical property of PoAgNPs is displayed in Figure 3a–d. To achieve stable and polydispersed PoAgNPs, we optimized various parameters by regulating the combination of silver nitrate salt and LA-1 supernatant, the stoichiometric proportion, the temperature, and the pH (Figure 3a–c). In Figure 3b, the pH 9 absorbance shift increased, indicating an increase in the synthesis of PoAgNPs. The alkaline pH will favor the reaction by an upsurge in the active constituents present in the extract. Temperature plays a crucial role in nanoparticle synthesis. In the present study, synthesis at 37 °C showed the maximum absorbance and a greater yield of nanoparticles (Figure 3c). The optimized synthesis parameters of quantity of culture extract (10 mL), concentration of silver nitrate (1 mM) and stoichiometric proportion of fungal extract: aqueous silver nitrate (10:90 mL), reaction temperature 37 °C, pH 9, incubation time of 24 h produced stable and polydispersed PoAgNPs.

#### 3.3.2. FT-IR Analysis of PoAgNPs

**Figure 4:**
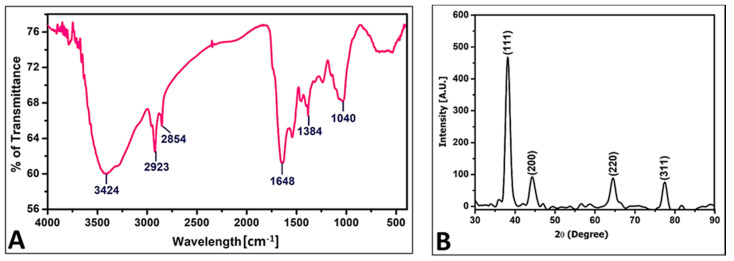
(**A**) FT-IR analysis of PoAgNPs. (**B**) XRD of PoAgNPs.

FT-IR analysis was performed to identify the possible functional groups that can act as reducing and capping agents in the synthesis of PoAgNPs. The FT-IR spectrum (Figure 4a) shows intensive peaks at 2923 cm^−1^, 2854 cm^−1^, 1648 cm^−1^, 1384 cm^−1^, and 1040 cm^−1^. The peak at 3424 cm^−1^ corresponds to the N–H amide of the protein, and the peak at 2923 cm^−1^ corresponds to the C–H stretch of the methylene groups present in the protein. The peak at 1648 cm^−1^ may be attributed to the –CO of the amide I band of the proteins; the peak at 1384 cm^−1^ was due to the C–N stretching vibrations of the aromatic amines.

#### 3.3.3. XRD Analysis of PoAgNPs

The crystalline nature of PoAgNPs was ascertained by using XRD analysis. The XRD spectrum for PoAgNPs is depicted in Figure 4b. From the XRD spectrum, four diffraction peaks were revealed at 27.71°, 32.20°, 46.21°, and 54.78°. These can be indexed as (111), (200), (220), and (311), which reflect the face-centered cubic crystalline nature of PoAgNPs.

#### 3.3.4. TEM and EDAX Analysis of PoAgNPs

**Figure 5:**
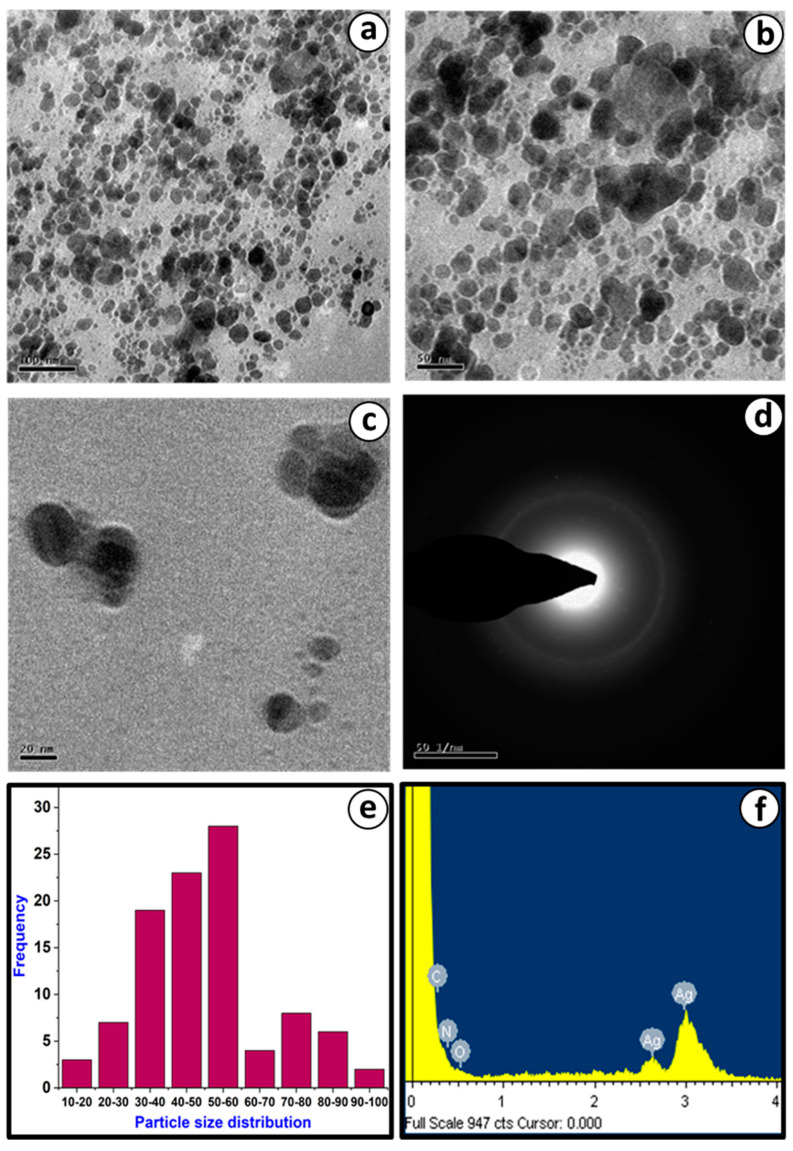
(**a**–**c**) TEM analysis of PoAgNPs. (**d**) SAED pattern of PoAgNPs. (**e**) Particle size histogram of PoAgNPs. (**f**) EDAX analysis of PoAgNPs.

TEM analysis revealed that PoAgNPs particles were randomly distributed with varying sizes and shapes (Figure 5a–d). Moreover, based on the TEM images’ analysis, the mean particle size of PoAgNPs was found to be 52.26 nm (Figure 5e). Elemental analysis of PoAgNPs was performed by EDAX, which displayed Ag as a major element present in the PoAgNPs (Figure 5f).

#### 3.3.5. Zeta Potential Analysis and DLS of PoAgNPs

**Figure 6:**
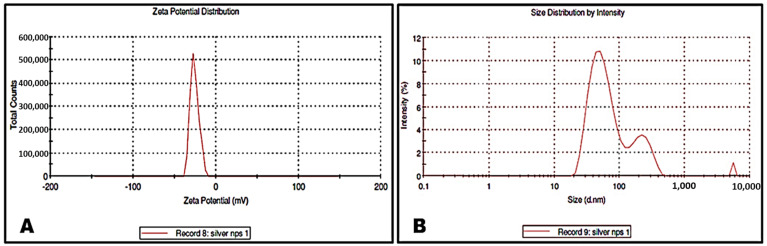
(**A**) Zeta potential of PoAgNPs. (**B**) Dynamic light scattering of PoAgNPs.

Surface charge and stability are the essential phenomena for application of nanoparticles in the field of biomedicine. A zeta potential analyzer evaluated the present study surface charge of PoAgNPs, and the obtained graph is shown in Figure 6a. From the graph, the surface charge of synthesized PoAgNPs was found to be −25.7 mV. The DLS measurement of PoAgNPs with an average size of 83.14 nm is shown in Figure 6b. The DLS scale is different because it gives the average size of the particles. That may also be due to the samples’ non-homogeneous dispersion of the sample.

### 3.4. Hemolytic Activity of PoAgNPs

**Figure 7:**
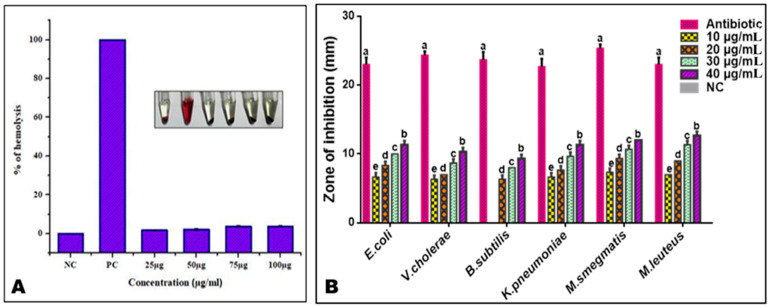
(**A**) Hemolytic activity of PoAgNPs. (**B**) Antibacterial activity of PoAgNPs (mean ± SE followed by different letters (a–e) within the same row were significantly different (Tukey’s test, *p* < 0.05)).

It is essential that the nanomaterial intended for medicinal use is mandatorily checked for risk assessment in toxicological aspects to ensure their biocompatibility. Our data showed that the synthesized PoAgNPs possessed a lower lysis profile than the positive control (Figure 7a).

### 3.5. Antibacterial Studies of PoAgNPs

#### 3.5.1. Antibacterial Activity

The antibacterial activity of PoAgNPs is presented in Figure 7b. Based on the results and zone of inhibition, PoAgNPs exhibited outstanding activity against the tested human pathogens. From the data, it is clear that the bactericidal activity progressed according to the dose level of PoAgNPs. The highest zone of inhibition was observed in the following order: *M. luteus > M.smegmatis > E. coli* > *K. pneumoniae > V. cholerae > B. subtilis.*

#### 3.5.2. MIC and MBC Analysis

The MIC and MBC values are shown in Table 2. The data inferred that MIC varies by the genera and species. The inhibitory concentration of PoAgNPs, except for *B. subtilis* (100 μg/mL), was found to be 25 µg/mL for Gram-positive bacterial pathogens (*M. smegmatis* and *M. luteus*), whereas for Gram-negative bacterial pathogens (*E. coli, V. cholerae,* and *K. pneumoniae*) it was found to be 50 µg/mL. However, the PoAgNPs significantly inhibited the growth of bacterial pathogens at low concentrations to high concentrations in an impressive manner. Furthermore, the MBC values portrayed the bactericidal effect of PoAgNPs with a value range from 100 to >100 µg/mL.

#### 3.5.3. Confirmation of MIC by Resazurin Dye Assay

**Figure 8:**
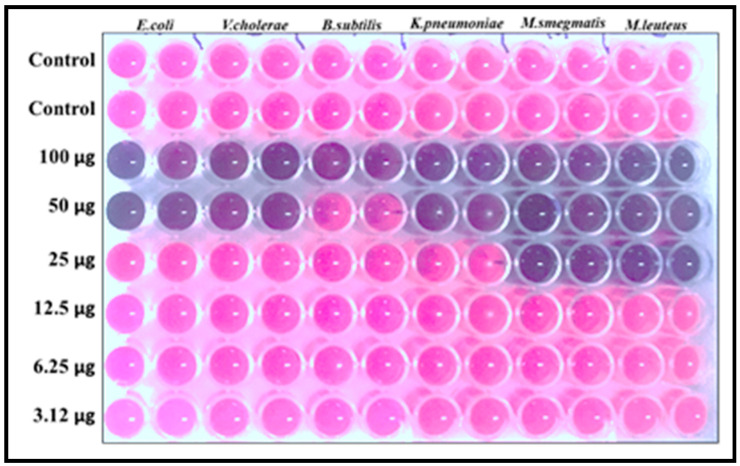
MIC values were confirmed by Resazurin dye assay.

Resazurin was used as an indicator in this study. In viable cells, oxidoreductases convert the resazurin salt to resorufin from blue to pink fluorescent. In the 96 well plates, according to the dose level of MIC, the color variation (blue to pink) can be visually observed in Figure 8. This indicated the viable and non-viable cells upon treatment of PoAgNPs.

#### 3.5.4. Fluorescence-Based Study of PoAgNPs against Bacterial Pathogens

**Figure 9:**
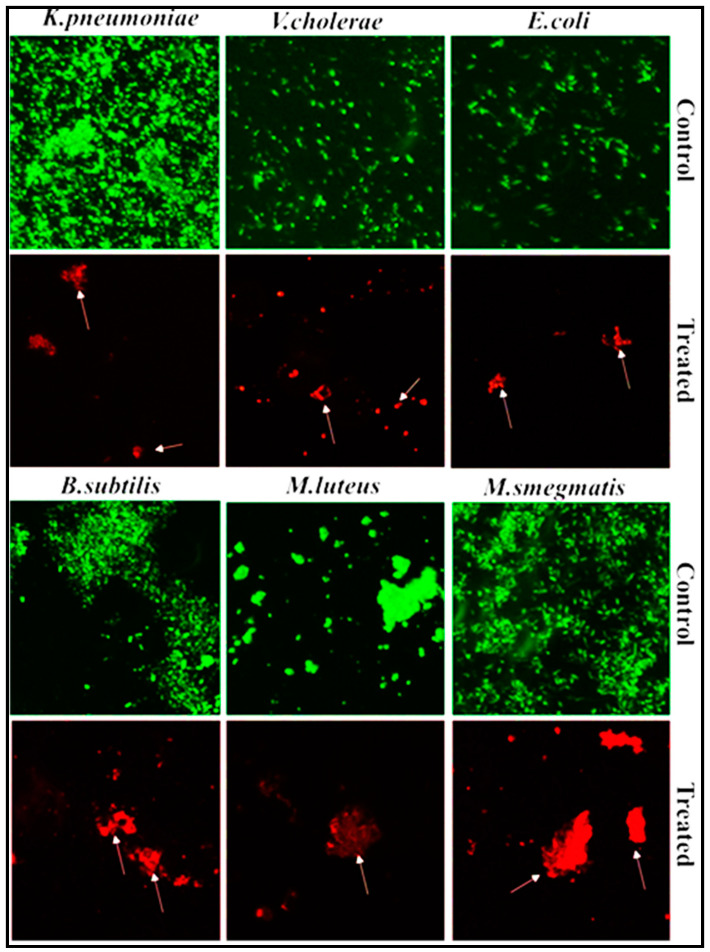
Fluorescence images of bacterial pathogens treated with MIC values of PoAgNPs. White arrows indicate the damaged bacterial cells.

The viability of bacterial cells exposed to PoAgNPs at MIC dosages (*E. coli* (50 µg/mL), *V. cholerae* (50 µg/mL), *B. subtilis* (100 µg/mL), *K. pneumoniae* (50 µg/mL), *M. smegmatis* (25 µg/mL), and *M. luteus* (25 µg/mL)) was observed using fluorescence microscopy. In the analysis, the live cells emitted green and dead cells emitted red fluorescence. This was because live cells uptake AO effectively and emit a green color, whereas dead cells uptake EB and emit red fluorescence (Figure 9).

### 3.6. In-Vitro Anticancer Activity of PoAgNPs

#### 3.6.1. MTT Assay

**Figure 10:**
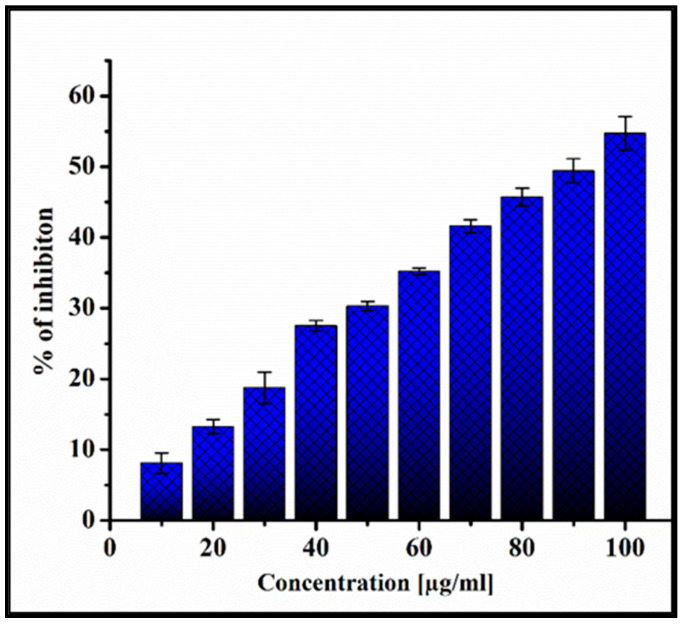
Cell proliferation analysis by MTT assay. Different concentrations of PoAgNPs (0 to 100 μg/mL) were used to treat MB-MDA-231 cancer cell lines. The proliferation rates were expressed as mean ± SD of the three experiments.

The in-vitro anticancer potential of the PoAgNPs was evaluated against human adenocarcinoma breast cancer (MDA-MB-231) cell lines by MTT assay. From the analysis, it was inferred that PoAgNPs trigger a pronounced inhibitory activity against the cell line with a gradual decline in cell viability in response to the concentration of PoAgNPs (10–100 µg/mL) with IC_50_ 91 µg/mL (as depicted in Figure 10).

#### 3.6.2. Dual Staining Assay (AO/EB)

**Figure 11:**
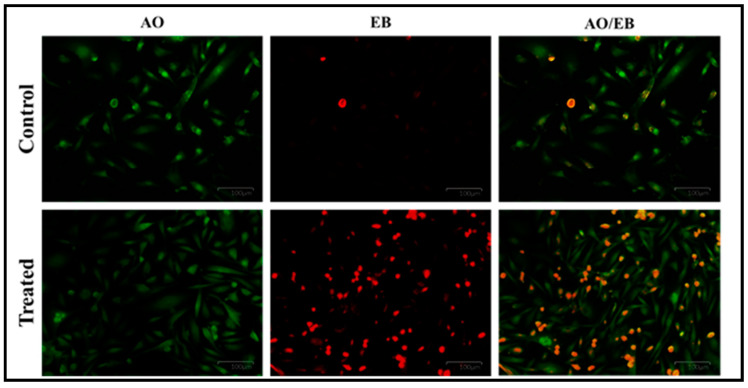
AO/EB dual staining apoptotic assay.

To authenticate that the IC_50_ concentration of PoAgNPs induced apoptosis, the cells were observed under AO/EB staining. The staining encountered the live and dead cells and differentiated them based on the color (live cells (green); dead cells (red); orange (late apoptotic cells)), as shown in Figure 11.

### 3.7. Larvicidal Activity of PoAgNPs against II and IV Instars Larvae of C. quinquefasciatus

**Figure 12:**
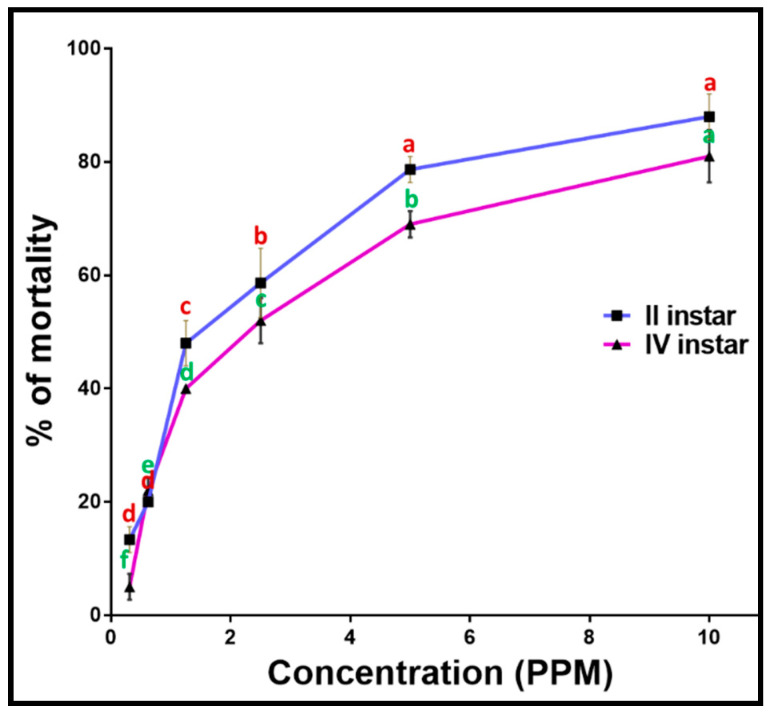
Larvicidal activity of PoAgNPs. Mean ± SE followed by different letters were significantly different (Tukey’s test, *p* < 0.05).

**Figure 13:**
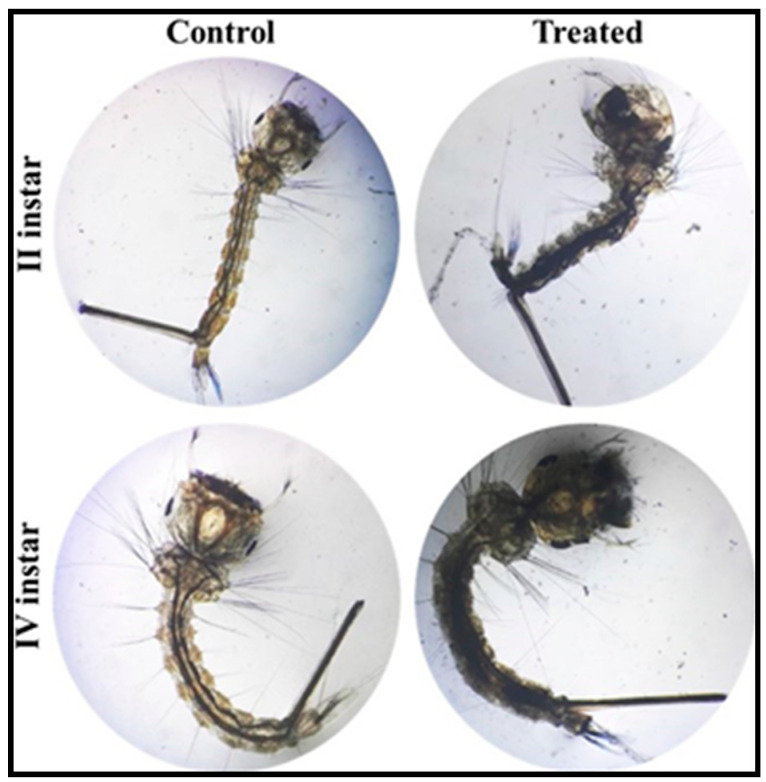
Light microscopic images of control- and PoAgNPs-treated mosquito larvae of *C. quinquefasciatus* with ×10 magnification.

Under laboratory conditions, we investigated the larvicidal efficacy of PoAgNPs against the II and IV larvae of *C. quinquefasciatus.* The larvicidal activity was performed with varying concentrations (2, 4, 6, 8, and 10 ppm) of PoAgNPs for 24 h exposure. After the treatment period, the larvae were analyzed for mortality. The obtained mortality data showed that PoAgNPs were toxic against the larvae of *C. quinquefasciatus* with significant LC_50_ values of 1.673 and 2.273 ppm (Figure 12; Table 3). The larvicidal activity of PoAgNPs depends on the mosquito larvae, genus, and species. Interestingly, in the present study, the minimal concentration of 1.6 ppm of PoAgNPs was required to kill 50% of mosquito larvae within a short period. This shows that PoAgNPs are a potentiated biocide to mitigate the eruption of mosquito larvae (Figure 13).

## 4. Discussion

In nature, a myriad of plants and microbes cohabit, which is exploited mainly for the welfare of human beings and the environment. In particular, the endophytic fungi produce many bioactive constituents with a potential for pharmacognostic applications [27]. This pipeline explored the nanobiotechnological potential of the endophytic fungi *P. oxalicum* strain LA-1 isolated from a medicinal plant. After subsequent isolation and identification, we screened the fractions of solvent extracts. Among the extracts, the highest percentage of compounds was observed in the methanolic extract compared with the other solvent extracts.

Noticeably, major pharmaceutical compounds such as pyrrolo[1, 2-a]pyrazine-1, 4-dione, and hexahydro-3-(2-methyl propyl) were noticed. As reported, these compounds have the potential for significant therapeutic applications [28,29,30]. Moreover, it is a well-established fact that the active hydroxyl or amine functional groups in bioactive constituents play a vital role in reducing metal ions. As the reaction proceeds by employing AgNO_3_ with the crude extract of *P. oxalicum* strain LA-1, a dark brown color was observed, indicating the synthesis of PoAgNPs.

Khan et al. [12] demonstrated that in UV–visible spectroscopic analysis of AgNPs, nanoparticles’ particle size, morphology, and compositions are directly proportional to the surface plasmon resonance. Herein, the optical properties of PoAgNPs were governed by the factor known as surface plasmon resonance, which generated absorbance spectra at 430 nm [31]. During the reaction, pH plays a crucial role, as a change in the electrical charges present in the culture filtrate substantially increases nanoparticles’ growth and yield by altering the reaction kinetics [32]. Our result was consistent with Singh et al. [30]. Qian et al. [33] demonstrated that alkaline pH favored the synthesis of silver nanoparticles when 1 mM silver nitrate was challenged with the cell-free filtrate of *E. nigrum*. The effect of temperature on the synthesis of silver nanoparticles was also explored in the present study. Even though temperatures at a higher range favored the formation of silver nanoparticles, we annotated that 37 °C was optimal for the stable formation of silver nanoparticles. These features were similar to the reports of Singh et al. [30]. Interestingly, in the FTIR spectrum of PoAgNPs, among the various peaks, a prominent peak located at 1040 cm^−1^ was found to be associated with the –C–OH of the phenols [34]. This was attributed to the fact that the proteins/alcohol/phenolic groups present in the culture filtrate were mainly responsible for reducing silver ions (Ag+) into nano-sized silver Ag (o) [35,36,37,38]. Additionally, in the XRD spectrum, four major diffraction peaks were located at 27.71°, 32.20°, 46.21°, and 54.78°, which could be indexed as (111), (200), (220), and (311), which reflected the face-centered cubic crystalline nature of PoAgNPs [39]. As a result, the XRD pattern obtained for the AgNPs showed the crystalline nature of AgNPs with an FCC (Face Centred Cubic) phase that corresponded to several previously reported studies on AgNPs synthesized by fungal extracts (Rafie et al. [37]; Asad et al. [38]). According to an earlier study [40], the peak of the silver ion was produced at 3 KeV, which could help in the reduction of Ag+ to Ag^0^.

The negative charge of synthesized PoAgNPs was mainly due to the culture extract of endophytic fungi containing biomolecules that modify nanoparticle surfaces with their anionic groups [41]. This negative charge bestows high stability and dispersity of NPs without aggregation owing to their strong repulsive forces. Furthermore, the observed hemolytic properties of PoAgNPs could be easily attributed to their size, surface chemistry, and physicochemical properties. The mechanism of PoAgNPs that induces hemolysis is by the interaction of AgNPs with the thiol group of protein and the phospholipids of RBC with a high affinity, which thereby collapses RBCs. Moreover, negatively charged silver ions interact with organic cations in the RBC membrane, which may also contribute to the hemolysis of RBC [42]. Hemolysis is a process of erythrocyte destruction with the release of hemoglobin in the environment [43]. According to the International Organization for Standardization/Technical Report 7406, the acceptable degree of hemolysis for bio-based products was 5% (Ruden et al. [44]). In our study, PoAgNPs demonstrated an adequate hemolysis rate, indicating its biocompatibility and suitability for biomedical applications. Our findings are consistent with the previous research of Sathish Kumar et al. [45].

In the present study, the inhibitory action of PoAgNPs was comparatively low in Gram-positive bacteria than in Gram-negative bacteria. This phenomenon was due to the presence of a thick peptidoglycan layer in which AgNPs could effectively stick to the cell wall of bacteria, thus preventing antibacterial action [46]. The bactericidal effect of AgNPs against pathogenic bacteria was achieved by attaching AgNPs to the bacterial cell surface and membrane, causing intracellular damage to the proteins, DNA, RNA, respiratory chain, and the metabolism, followed by induction of oxidative stress (ROS generation) and, finally, cell death [47]. Hence, we speculated that PoAgNPs may exhibit the above fourth mechanism against the tested pathogens.

The in-vitro cytotoxicity exhibited an effective growth inhibitory activity in human adenocarcinoma breast cancer (MDA-MB-231) cell lines by MTT assay. The PoAgNPs triggered the toxicity by inducing apoptosis in a dose-dependent manner. The mechanistic action of PoAgNPs is executed by induction of ROS, followed by disruption of intracellular organelles, which eventually leads to apoptosis [48]. The color variation is based on the uptake of dyes. Live cells uptake the low molecular weight dye AO, whereas dead cells uptake the high molecular weight dye EB. This phenomenon occurs when cells undergo apoptosis; thus, the cell membrane blebs and leaks, nuclei condense, and DNA fragmentation can be observed [49].

Apart from the antibacterial properties, the biogenic AgNPs have been reported to hold excellent larvicidal properties against disease-transmitting mosquito vectors [50]. Prabakaran and colleagues recorded that silver nanoparticles from *Beauveria bassiana* against different larval stages of *Culex* sp. had significant mortality effects, similar to what we found in our current study [51]. Our data clearly showed the possible mechanism of AgNPs against the larvae by breaching the exoskeleton of *C. quinquefasciatus* mosquito larvae that binds with the phosphorous/sulfur of DNA and protein, which results in the blockade of transcription and translation followed by the internal collapse of cell organelles and, finally, cell death [52].

## 5. Conclusions

To summarize, we documented the synthesis of PoAgNPs using a culture filtrate of the endophytic fungi *P. oxalicum* LA-1 strain. We exclusively characterized the PoAgNPs through UV–vis spectroscopy, FTIR, XRD, and TEM analyses. The synthesized PoAgNPs inhibited human pathogenic bacterial growth, which depicts its potential as an antibacterial agent. PoAgNPs effectively demonstrated the increased inhibitory activity in human adenocarcinoma breast cancer (MDA-MB-231) cell lines by MTT assay with the IC_50_ concentration of 91 µg/mL. Moreover, larvicidal screening showed that PoAgNPs are an effective biocide against the disease-transmitting *C. quinquefasciatus*. Finally, PoAgNPs unveiled significantly less RBC lysis, which denotes its biocompatibility and possibility for medicinal use. Overall, we believe that a sustainable approach was developed to synthesize versatile PoAgNPs with improved physicochemical properties. The synthesized nanomaterials can be developed as an antibacterial/larvicide agent after being subject to in vitro and in vivo pre-clinical studies. Based on the obtained results, we conclude that the synthesized PoAgNPs could act as a potent antibacterial/antitumor agent after completing the pre-clinical and clinical studies.

## Figures and Tables

**Table 1:** GC-MS analysis for methanolic fungal extract.

S. No.	Retention Time	% of Total	M.W.	M. Formula	Compound Name
1	11.394	3.111	310	C_16_H_29_F_3_O_2_	3-trifluoroacetoxytetradecane
2	16.801	1.446	238	C_16_H_30_O	E-14-hexadecenal
3	18.117	3.3	206	C_14_H_22_O	Phenol, 2,4-bis(1,1-dimethylethyl)-
4	19.333	2.771	238	C_16_H_30_O	E-14-hexadecenal
5	20.963	3.225	186	C_12_H_10_O_2_	1-naphthalene carboxylic acid, methyl ester
6	21.274	1.525	238	C_16_H_30_O	E-14-hexadecenal
7	21.642	1.825	242	C_15_H_30_O_2_	Pentadecanoic acid
8	21.32	1.211	210	C_11_H_18_N_2_O_2_	Pyrrolo[1,2-a]pyrazine-1,4-dione, hexahydro-3-(2-methylpropyl)-
9	22.46	2.082	210	C_11_H_18_N_2_O_2_	Pyrrolo[1,2-a]pyrazine-1,4-dione, hexahydro-3-(2-methylpropyl)-
10	22.522	1.784	496	C_27_H_52_O_4_Si_2_	9,12,15-octadecatrienoic acid
11	22.744	5.481	210	C_11_H_18_N_2_O_2_	Pyrrolo[1,2-a]pyrazine-1,4-dione, hexahydro-3-(2-methylpropyl)-
12	22.872	4.745	210	C_11_H_18_N_2_O_2_	Pyrrolo[1,2-a]pyrazine-1,4-dione, hexahydro-3-(2-methylpropyl)-
13	22.922	2.623	210	C_11_H_18_N_2_O_2_	Pyrrolo[1,2-a]pyrazine-1,4-dione, hexahydro-3-(2-methylpropyl)-
14	23.091	5.819	210	C_11_H_18_N_2_O_2_	Pyrrolo[1,2-a]pyrazine-1,4-dione, hexahydro-3-(2-methylpropyl)-
15	23.541	2.329	256	C_16_H_32_O_2_	n-hexadecanoic acid
16	25.431	4.221	238	C_16_H_30_O	E-14-hexadecenal
17	25.896	2.479	226	C_12_H_22_N_2_O_2_	2,5-piperazinedione, 3,6-bis(2-methylpropyl)-
18	27.464	2.952	266	C_18_H_34_O	5-octadecenal
19	29.421	1.632	210	C_11_H_18_N_2_O_2_	Pyrrolo[1,2-a]pyrazine-1,4-dione, hexahydro-3-(2-methylpropyl)-
20	31.769	8.132	281	C_18_H_35_NO	9-octadecenamide, (Z)-

**Table 2:** MIC and MBC values for PoAgNPs against bacterial pathogens.

Bacterial Pathogens	Gram-Negative			Gram-Positive		
	*E. coli*	*V. cholerae*	*K. pneumoniae*	*B. subtilis*	*M. smegmatis*	*M. luteus*
MIC (µg/mL)	50	50	50	100	25	25
MBC (µg/mL)	>100	100	>100	>100	100	>100

**Table 3:** LC_50_ and LC_90_ values of PoAgNPs against *Culex mosquito* at different stages.

Larvae Stages	LC_50_ (95% Fiducial Limits (LCL-UCL))(PPM)	LC_90_ (95% Fiducial Limits (LCL-UCL))(PPM)	Regression Equations	R Value
II instar	1.673 (1.521–1.840)	10.85 (9.12–13.27)	27.688X + 7.138	0.82
IV instar	2.273 (2.059–2.515)	16.24 (13.29–20.56)	22.773X + 6.808	0.85

SEM: standard error mean, R: regression coefficient, LC_50_: lethal concentration that kills 50% of the treated larvae in micrograms per milliliter, LC_90_: lethal concentration that kills 90% of treated larvae in micrograms per milliliter.

## Data Availability

Data available on request due to restrictions eg privacy or ethical.

## References

[1] Meera R. (2012). Nanostructures and their applications. Recent Res. Sci. Technol..

[2] Sekhon B. (2014). Nanotechnology in agri-food production: An overview. Nanotechnol. Sci. Appl..

[3] Ejad R.M., Karimi S., Iravani S., Varma R.S. (2016). Plant-derived nanostructures: Types and applications. Green Chem..

[4] Marassi V., Di Cristo L., Smith S., Ortelli S., Blosi M., Costa A.L., Reschiglian P., Volkov Y., Prina-Mello A. (2018). Silver nanoparticles as a medical device in healthcare settings: A five-step approach for candidate screening of coating agents. R. Soc. Open Sci..

[5] Ferlay J., Colombet M., Soerjomataram I., Mathers C., Parkin D.M., Pineros M., Znaor A., Bray F. (2019). Estimating the global cancer incidence and mortality in 2018: GLOBOCAN sources and methods. Int. J. Cancer.

[6] Curigliano G., Criscitiello C. (2014). Successes and limitations of targeted cancer therapy in breast cancer. Prog. Tumor Res..

[7] Kumari P., Ghosh B., Biswas S. (2016). Nanocarriers for cancer-targeted drug delivery. J. Drug Target..

[8] Mayo Clinic (2018). Chemotherapy for Breast Cancer—Mayo Clinic. https://www.mayoclinic.org/tests-procedures/chemotherapy-for-breast-cancer/about/pac-20384931.

[9] Tang X., Cai S., Zhang R., Liu P., Chen H., Zheng Y., Sun L. (2013). Paclitaxel-loaded nanoparticles of star-shaped cholic ac-id-core PLA-TPGS copolymer for breast cancer treatment. Nanoscale Res. Lett..

[10] Sonawnae A., Jena P., Mohanty S., Mallick R., Jacob B. (2012). Toxicity and antibacterial assessment of chitosan-coated silver nanoparticles on human pathogens and macrophage cells. Int. J. Nanomed..

[11] Zhang L., Gu F.X., Chan J.M., Wang A.Z., Langer R.S., Farokhzad O.C. (2008). Nanoparticles in medicine: Therapeutic applications and developments. Clin. Pharmacol. Ther..

[12] Khan I., Saeed K., Khan I. (2019). Nanoparticles: Properties, applications and toxicities. Arab. J. Chem..

[13] Iravani S., Korbekandi H., Mirmohammadi S., Zolfaghari B. (2015). Synthesis of silver nanoparticles: Chemical, physical and biological methods. Res. Pharm. Sci..

[14] El-Nour K.M.A., Eftaiha A., Al-Warthan A., Ammar R.A. (2010). Synthesis and applications of silver nanoparticles. Arab. J. Chem..

[15] Li X., Xu H., Chen Z.-S., Chen G. (2011). Biosynthesis of Nanoparticles by Microorganisms and Their Applications. J. Nanomater..

[16] Durán N., Marcato P.D., Alves O.L., De Souza G.I.H., Esposito E. (2005). Mechanistic aspects of biosynthesis of silver nanoparticles by several Fusarium oxysporum strains. J. Nanobiotechnol..

[17] Moghaddam A.B., Namvar F., Moniri M., Tahir P.M., Azizi S., Mohamad R. (2015). Nanoparticles Biosynthesized by Fungi and Yeast: A Review of Their Preparation, Properties, and Medical Applications. Molecules.

[18] Strobel G., Daisy B., Castillo U., Harper J. (2004). Natural Products from Endophytic Microorganisms. J. Nat. Prod..

[19] Aly A.H., Debbab A., Kjer J., Proksch P. (2010). Fungal endophytes from higher plants: A prolific source of phytochemicals and other bioactive natural products. Fungal Divers..

[20] Prabukumar S., Sathishkumar G., Rajkuberan C., Gobinath C., Asad S., Hodhod M.S., Fuad A., Sivaramakrishnan S. (2018). Isolation of limonoid compound (Hamisonine) from endophytic fungi Penicillium oxalicum LA-1 (KX622790) of Limonia acidissima L. for its larvicidal efficacy against LF vector, Culex quinquefasciatus (Diptera: Culicidae). Environ. Sci. Pollut. Res..

[21] Rajkuberan C., Sudha K., Sathishkumar G., Sivaramakrishnan S. (2015). Antibacterial and cytotoxic potential of silver nanopar-ticles synthesized using latex of *Calotropis gigantea* L. Spectrochim. Acta A Mol. Biomol. Spectrosc..

[22] Dong Y., Yang Y., Wei Y., Gao Y., Jiang W., Wang G., Wang D. (2020). Facile synthetic nano-curcumin encapsulated Bio-fabricated nanoparticles induces ROS-mediated apoptosis and migration blocking of human lung cancer cells. Process. Biochem..

[23] Priyadarshini S., Sonsudin F., Mainal A., Yahya R., Gopinath V., Vadivelu J., Alarjani K.M., Al Farraj D.A., Yehia H.M. (2021). Phytosynthesis of biohybrid nano-silver anchors enhanced size dependent photocatalytic, antibacterial, anticancer properties and cytocompatibility. Process. Biochem..

[24] Wiegand I., Hilpert K., Hancock R.E.W. (2008). Agar and Broth Dilution Methods to Determine the Minimal Inhibitory Concen-tration (MIC) of Antimicrobial Substances. Nat. Protoc..

[25] Manikandan R., Anjali R., Beulaja M., Prabhu N.M., Koodalingam A., Saiprasad G., Chitra P., Arumugam M. (2019). Synthesis, characterization, anti-proliferative and wound healing activities of silver nanoparticles synthesized from Caulerpa scalpelli-formis. Process Biochem..

[26] Rajkuberan C., Prabukumar S., Muthukumar K., Sathishkumar G., Sivaramakrishnan S. (2018). Carica papaya (Papaya) latex: A new paradigm to combat against dengue and filariasis vectors Aedes aegypti and Culex quinquefasciatus (Diptera: Culicidae). 3 Biotech.

[27] Porras-Alfaro A., Bayman P. (2011). Hidden Fungi, Emergent Properties: Endophytes and Microbiomes. Annu. Rev. Phytopathol..

[28] Kiran G.S., Priyadharsini S., Sajayan A., Ravindran A., Selvin J. (2018). An antibiotic agent pyrrolo[1,2-a]pyrazine-1,4-dione,hexahydro isolated from a marine bacteria Bacillus tequilensis MSI45 effectively controls multi-drug resistant Staphylococcus aureus. RSC Adv..

[29] Sheoran N., Nadakkakath A.V., Munjal V., Kundu A., Subaharan K., Venugopal V., Rajamma S., Eapen S.J., Kumar A. (2015). Genetic analysis of plant endophytic Pseudomonas putida BP25 and chemo-profiling of its antimicrobial volatile organic compounds. Microbiol. Res..

[30] Singh D., Rathod V., Ninganagouda S., Herimath J., Kulkarni P. (2014). Optimization and Characterization of Silver Nanoparticle by Endophytic Fungi *Penicillium* sp. Isolated from *Curcuma longa* (Turmeric) and Application Studies against MDR E. coli and S. aureus. Bioinorg. Chem. Appl..

[31] Amendola V., Bakr O.M., Stellacci F. (2010). A Study of the Surface Plasmon Resonance of Silver Nanoparticles by the Discrete Dipole Approximation Method: Effect of Shape, Size, Structure, and Assembly. Plasmonics.

[32] Netala V.R., Kotakadi V.S., Bobbu P., Gaddam S.A., Tartte V. (2016). Endophytic fungal isolate mediated biosynthesis of silver nanoparticles and their free radical scavenging activity and anti-microbial studies. 3 Biotech.

[33] Qian Y., Yu H., He D., Yang H., Wang W., Wan X., Wang L. (2013). Biosynthesis of silver nanoparticles by the endophytic fungus Epicoccum nigrum and their activity against pathogenic fungi. Bioprocess Biosyst. Eng..

[34] Jain S., Mehata M.S. (2017). Medicinal Plant Leaf Extract and Pure Flavonoid Mediated Green Synthesis of Silver Nanoparticles and their Enhanced Antibacterial Property. Sci. Rep..

[35] Litvin V.A., Minaev B.F. (2013). Spectroscopy study of silver nanoparticles fabrication using synthetic humic substances and their antimicrobial activity. Spectrochim. Acta Part A Mol. Biomol. Spectrosc..

[36] Jaidev L.R., Narasimha G. (2010). Fungal mediated biosynthesis of silver nanoparticles, characterization and antimicrobial activity. Colloids Surf. B Biointerfaces.

[37] El-Rafie M., Shaheen T., Mohamed A., Hebeish A. (2012). Bio-synthesis and applications of silver nanoparticles onto cotton fabrics. Carbohydr. Polym..

[38] Syed A., Saraswati S., Kundu G.C., Ahmad A. (2013). Biological synthesis of silver nanoparticles using the fungus *Humicola* sp. and evaluation of their cytoxicity using normal and cancer cell lines. Spectrochim. Acta Part A Mol. Biomol. Spectrosc..

[39] Singh T., Jyoti K., Patnaik A., Singh A., Chauhan R., Chandel S.S. (2017). Biosynthesis, characterization and antibacterial activity of silver nanoparticles using an endophytic fungal supernatant of *Raphanus sativus*. J. Genet. Eng. Biotechnol..

[40] Masum M.I., Siddiqa M.M., Ali K.A., Zhang Y., Abdallah Y., Ibrahim E., Qiu W., Yan C., Li B. (2019). Biogenic Synthesis of Silver Nanoparticles Using *Phyllanthus emblica* Fruit Extract and Its Inhibitory Action Against the Pathogen Acidovorax oryzae Strain RS-2 of Rice Bacterial Brown Stripe. Front. Microbiol..

[41] Ashokraja C., Sakar M., Balakumar S. (2017). A perspective on the hemolytic activity of chemical and green-synthesized silver and silver oxide nanoparticles. Mater. Res. Express.

[42] Tang S., Zheng J. (2018). Antibacterial Activity of Silver Nanoparticles: Structural Effects. Adv. Healthc. Mater..

[43] Rai M.K., Deshmukh S.D., Ingle A.P., Gade A.K. (2012). Silver nanoparticles: The powerful nano weapon against multi-drug-resistant bacteria. J. Appl. Microbiol..

[44] Ruden S., Hilpert K., Berditsch M., Wadhwani P., Ulrich A.S. (2009). Synergistic Interaction between Silver Nanoparticles and Membrane-Permeabilizing Antimicrobial Peptides. Antimicrob. Agents Chemother..

[45] Gnanasekar S., Murugaraj J., Dhivyabharathi B., Krishnamoorthy V., Jha P.K., Seetharaman P., Sivaperumal S. (2018). Anti-bacterial and cytotoxicity effects of biogenic palladium nanoparticles synthesized using fruit extract of *Couroupita guianensis* Aubl. J. Appl. Biomed..

[46] Benelli G., Caselli A., Canale A. (2017). Nanoparticles for mosquito control: Challenges and constraints. J. King Saud Univ. Sci..

[47] Golubeva O.Y., Shamova O.V., Orlov D.S., Pazina T.Y., Boldina A.S., Kokryakov V.N. (2010). Study of antimicrobial and he-molytic activities of silver nanoparticles prepared by chemical reduction. Glass Phys. Chem..

[48] Gurunathan S., Jegadeesh R., Sri N.A.M., Priscilla A.J., Sabaratnam V. (2013). Green synthesis of silver nanoparticles using Ganoderma neo-japonicum Imazeki: A potential cytotoxic agent against breast cancer cells. Int. J. Nanomed..

[49] Krishnaraj C., Muthukumaran P., Ramachandran R., Balakumaran M., Kalaichelvan P. (2014). Acalypha indica Linn: Biogenic synthesis of silver and gold nanoparticles and their cytotoxic effects against MDA-MB-231, human breast cancer cells. Biotechnol. Rep..

[50] Patil S., Chandrasekaran R. (2020). Biogenic nanoparticles: A comprehensive perspective in synthesis, characterization, application and its challenges. J. Genet. Eng. Biotechnol..

[51] Prabakaran K., Ragavendran C., Natarajan D. (2016). Mycosynthesis of silver nanoparticles from Beauveria bassiana and its larvi-cidal, antibacterial, and cytotoxic effect on human cervical cancer (HeLa) cells. RSC Adv..

[52] Chen L.Q., Fang L., Ling J., Ding C.Z., Kang B., Huang C.Z. (2015). Nanotoxicity of Silver Nanoparticles to Red Blood Cells: Size Dependent Adsorption, Uptake, and Hemolytic Activity. Chem. Res. Toxicol..

